# Long-term efficacy of surgical resection with or without adjuvant therapy for treatment of secondary glioblastoma in adults

**DOI:** 10.1093/noajnl/vdaa098

**Published:** 2020-08-21

**Authors:** Ruoyu Huang, Guanzhang Li, Yiming Li, Yinyan Wang, Pei Yang, Chuanbao Zhang, Zheng Wang, Dabiao Zhou, Wei Zhang, Zhong Zhang, Tao Jiang

**Affiliations:** 1 Department of Molecular Neuropathology, Beijing Neurosurgical Institute, Capital Medical University, Beijing, China; 2 Department of Neurosurgery, Beijing Tiantan Hospital, Capital Medical University, Beijing, China; 3 Center of Brain Tumor, Beijing Institute for Brain Disorders, Beijing, China; 4 China National Clinical Research Center for Neurological Diseases, Beijing, China; 5 Chinese Glioma Genome Atlas Network (CGGA) and Asian Glioma Genome Atlas Network (AGGA), Beijing, China

**Keywords:** adjuvant therapy, extent of resection, IDH1 mutation, secondary GBM

## Abstract

**Background:**

There are limited studies on treatment strategies and associated clinical outcomes in patients with secondary glioblastoma (sGBM). We sought to investigate the prognostic factors and treatment decisions in a retrospective cohort of patients with sGBM.

**Methods:**

One hundred and seventy-one patients with sGBM who met the screening criteria were included in this study. Kaplan–Meier survival analysis and Cox survival analysis were used to detect prognostic factors. R (v3.5.0) and SPSS software (v25.0, IBM) were used to perform statistical analyses.

**Results:**

The median overall survival was 303 days (range 23–2237 days) and the median progression-free survival was 229 days (range 33–1964 days) in patients with sGBM. When assessing the relationship between adjuvant treatment outcome and extent of resection (EOR), the results showed that patients underwent gross total resection can benefit from postoperative radiotherapy and chemotherapy, but not in patients underwent subtotal resection. In addition, we also found that aggressive adjuvant therapy can significantly improve clinical outcomes of IDH1-mutated patients but no significant prognostic value for IDH1-wildtyped patients. The univariate Cox regression analyses demonstrated that EOR, adjuvant therapy, and postoperative Karnofsky Performance Scores were prognostic factors for patients with sGBM, and multivariate COX analysis confirmed that adjuvant therapy and EOR were independent prognostic factors.

**Conclusions:**

For patients with sGBM, aggressive postoperative adjuvant therapy after gross total resection was recommended. However, we did not detect a benefit in IDH1-wildtype patients in our cohort.

**Key Points**

- EOR and adjuvant treatment were independent prognostic factors for sGBM patients.
- GTR combined with adjuvant therapy was the best treatment strategy for sGBM patients.
- The IDH1-wildtyped patients could not benefit from GTR and adjuvant therapy.

**Importance of the Study**

Secondary glioblastoma was a special kind of glioblastoma and had many significant differences with primary glioblastoma. However, limited studies focus on treatment strategies and associated clinical outcomes in patients with secondary glioblastoma. In this study, we found that gross total resection combined with aggressive postoperative adjuvant therapy was the best choice for patients with secondary glioblastoma. But for IDH1-wildtyped patients, exploring new treatment options is essential to improve prognosis of patients. The results mentioned above have a directive and practical importance to the clinical treatment of secondary glioblastoma.

Glioblastoma (GBM, WHO Grade IV) is the most common malignant primary brain tumor among adults.^1,2^ Based on clinical history GBMs are further classified into 2 subtypes: “primary glioblastoma (pGBM)” arising de novo without detectable malignant precursor lesion and “secondary glioblastoma (sGBM)” evolving from previously lower-grade (WHO Grade II or III) gliomas.^3,4^ Despite histologically indistinguishable, they show distinctive epidemiological, molecular, and genetic profile.^5^ The age of patients with a clinical diagnosis of sGBM is on average 10–20 years younger than those with pGBM.^6,7^ In addition, only sGBM but not pGBM show high-frequency IDH1/2 mutations as well as low-frequency epidermal growth factor receptor (EGFR) amplification.^7–9^ The clinical course is substantially longer in patients with sGBM, indicative of low malignancy of secondary tumors.^9,10^ Overall, pGBM and sGBM are distinct tumor entities and may require different treatment strategies.

Although numerous studies have addressed therapy in lower-grade gliomas and GBMs, there is limited information on prognostic factors and treatment decisions in patients with sGBM. Previously reported prognosis factors for sGBM include IDH mutation, MGMT promoter methylation, 1p/19q codeletion, frontal localization, the extent of resection (EOR), and postoperative Karnofsky Performance Scores (KPS).^10–12^ However, the series reported in the literature have a relatively small number of patients. The results are quite different among different studies. These factors hinder the clinical application of previous findings.

The Chinese Glioma Genome Atlas (CGGA) was founded to promote clinical research on the management of gliomas and establish a standardized treatment system for neuro-oncology. The aim of this study was to evaluate the impact of molecular pathology and clinical treatment on survival in a retrospective cohort of sGBM based on our database.

## Materials and Methods

### Selection of Patients

Secondary GBM patients were screened out in our database according to the following criteria: (1) age between 18 and 65 years at time of surgery; (2) diagnosed as sGBM as first time (the pathological diagnosis was GBM, and the previous pathological diagnosis was low-grade glioma or anaplastic glioma); (3) availability of clinical information, postoperative treatment information, and follow-up information in the period March 2006 to May 2018; (4) no other significant systemic diseases or malignancies during follow-up. Finally, 171 sGBM patients were included in this study (Supplementary Figure S1). This study was approved by the Institutional Review Boards of Beijing Tiantan Hospital, and written informed consents were obtained from all patients.

### Surgical and Adjuvant Treatment

Surgery was performed using a surgical microscope under the guidance of intraoperative neuronavigation. The extent of surgery was assessed from surgical reports and MRI within 72 h after surgery. ≥95% resection was defined as gross total removal and 50%–95% resection was defined as a subtotal removal. Postoperative adjuvant treatment was performed based on the imaging results and the patient’s condition. The adjuvant treatment in this study included temozolomide chemotherapy and/or radiotherapy. In this study, for low-grade glioma (WHO Grade II), the “extent of resection” was defined as “resection of both enhancing and non-enhancing disease.” But for high-grade glioma (WHO Grades III and IV), the “extent of resection” was defined as “complete resection of enhancing disease.”

### Molecular Testing

Molecular testing was performed at the Molecular Pathology Testing Center of Beijing Neurosurgical Institute.

IDH1/2 mutation status was assessed by pyrosequencing. The primers 5′-GCTTGTGAGTGGATGGGTAAAAC-3′, 5′-BiotinTTGCCAACATGAC TTACTTGATC-3′ for IDH1 and 5′-ATCCTGGGGGGGACTGTCTT-3′, 5′-Biotin-CTCTCCACCCTGGCCT ACCT-3′ for IDH2 were used for polymerase chain reaction (PCR) amplification, and the primers 5′-TGGATGGGTAAAACCT-3′ for IDH1 and 5′-AGCCCATCACCATTG-3′ for IDH2 were used for pyrosequencing. All the type of IDH1 mutation was R132H.

The chromosomal status of 1p and 19q was assessed by fluorescent in situ hybridization (FISH) with locus-specific probes for 1p36 and 19q13 FISH Probe Kit (Abbott Molecular). More than 25% of counted nuclei presented 1 target (orange) signal and 2 references (green) signals were considered as 1p or 19q deleted.

MGMT promoter status was assessed by methylation-specific PCR as previously described.^13^

### Statistical Analysis

Statistical analyses and drawing were performed by software environment R (v3.5.0) and SPSS software (v25.0, IBM). The Student’s *t*-test was used to assess differences between 2 continuous variables and the Chi-square test was performed to validate the differences in categorical variables. The log-rank test was used to assess the survival differences between groups in Kaplan–Meier analysis. *P* values lower than .05 were considered statistically significant.

## Result

### Baseline Characteristics of Patients

The baseline characteristics and their survival values are shown in Table 1. Among these patients, there were 113 men and 58 women. The median age of all patients was 41 years (range 18–65 years). A total of 79 patients were younger than or equal to 40 years, and 92 patients were older than 40 years. The median follow-up time is 460 days (range 142–2558 days). Up to last follow-up date, 143 patients have died while 18 patients are still alive. Besides, 10 patients were lost to follow-up.

**Table 1.** Patient Information

Characteristic	No. of patients	*P* value (log-rank test)	
		OS	PFS
Gender		.2201	.3562
Male	113		
Female	58		
Age (years)		.1676	.7100
Range	18–65		
Median	41		
≤40	79		
>40	92		
Resection		<.0001	.0443
Total	92		
Subtotal	79		
Postoperative treatment		<.0001	<.0001
Chemo + Radio	34		
Chemo	77		
Observe	36		
Others and NA	24		
IDH1 status		.0566	.0314
Mutation	67		
Wildtype	41		
NA	63		
MGMT promoter		.6266	.9303
Methylation	34		
Unmethylation	20		
NA	117		
1p19q		.1291	.2883
Codel	9		
Intact	57		
KPS			
Preoperative KPS	165	.0631	.3823
Postoperative KPS	171	.0393	.0353

In the surgical procedure, the accurate resection degree was obtained in 171 patients. Eleven patients with uncertain resection information were excluded from the analysis of tumor resection. Ninety-two patients (53.8%) underwent total resection and 79 patients (46.2%) underwent subtotal resection. Among all sGBM patients, 148 patients had complete information about postoperative adjuvant therapy. Thirty-four patients (23%) received radiotherapy and chemotherapy after surgery, and 77 patients (52%) received chemotherapy only. In addition, 36 patients (24.3%) did not receive any postoperative adjuvant therapy. One patient who received radiotherapy only after surgery was excluded from the analysis of postoperative therapy.

According to the results of molecular pathology, 108 patients had information on IDH1 mutation status. Among them, tumors of 67 patients were IDH1 mutation while 41 patients were IDH1 wildtype. In addition, tumors of 34 patients had MGMT promoter methylation and 20 patients were MGMT promoter unmethylation. We also identified that tumors of 9 patients had 1p19q codeletion and 57 patients did not have this condition. Other patients who did not undergo relevant molecular pathology tests or the information was not clear were excluded from the survival analysis of corresponding molecular pathology.

### Treatment Outcomes for Patients With Different EOR and Postoperative Adjuvant Therapy

**Figure 1.**
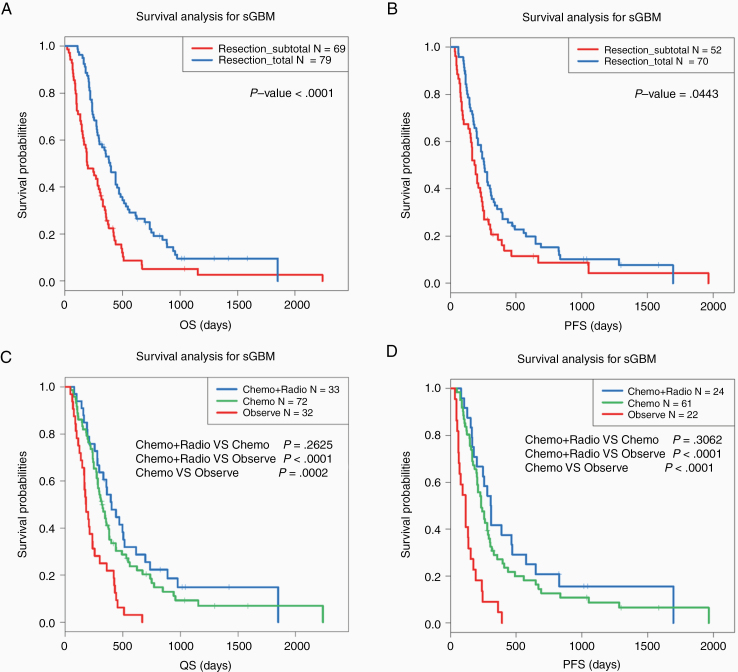
Kaplan–Meier survival curve analysis for sGBM patients with different surgical and adjuvant treatment. (A and B) OS and PFS among sGBM patients with different EOR. (C and D) OS and PFS among sGBM patients with different postoperative adjuvant therapy.

Overall survival (OS) status was available for 158 patients and progression-free survival (PFS) status was available for 122 patients. The median OS of all patients was 303 days (range 23–2237 days), and the median PFS was 229 days (range 33–1964 days). The association between the extent of surgical resection and the clinical outcomes of patients with sGBM was investigated by Kaplan–Meier survival curve analysis. Kaplan–Meier curves indicated that there was significant difference in OS (*P* < .0001, log-rank test; Table 1) and PFS (*P* = .0443, log-rank test; Table 1) between patients underwent total resection and subtotal tumor resection (Figure 1A and B). Further analysis found that there was no significant difference in clinicopathological and molecular features between patients in different tumor resection groups (Supplementary Table S1).

**Figure 2.**
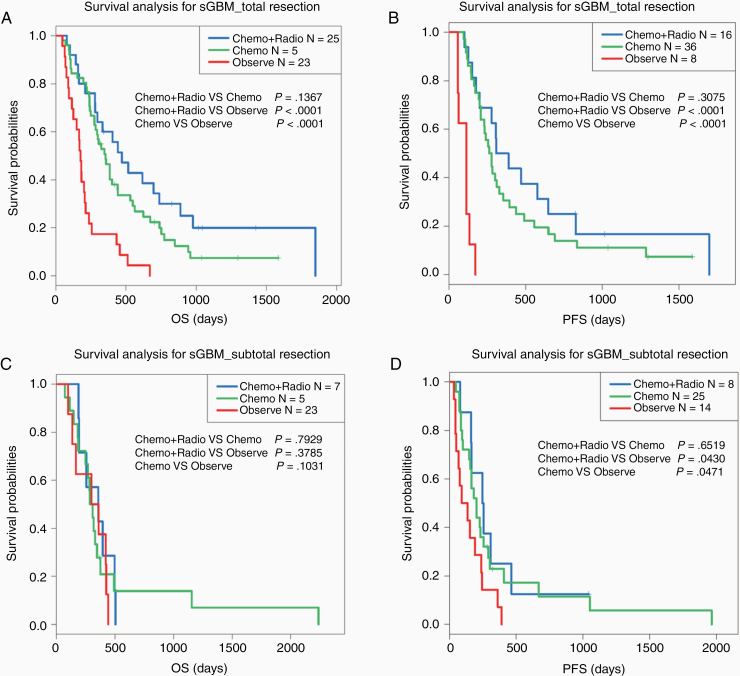
Kaplan–Meier survival curve analysis for sGBM patients with different treatment strategies. (A and B) OS and PFS among sGBM patients with different postoperative adjuvant therapy after gross total resection. (C and D) OS and PFS among sGBM patients with different postoperative adjuvant therapy after subtotal resection.

Then, we further explored the impact of different adjuvant treatments after surgery on the clinical outcomes of sGBM patients. The results showed that patients who did not receive any postoperative treatment had the worst prognosis compared with those who received radiochemotherapy or chemotherapy alone (Figure 1C and D). To further explore the effect of surgical resection on the efficacy of postoperative adjuvant therapy. We divided sGBM patients into 2 subgroups according to the EOR. The Kaplan–Meier curve analysis showed that postoperative radiochemotherapy or chemotherapy can significantly improve prognosis in patients who underwent total resection (Figure 2A and B). However, for patients underwent subtotal resection, postoperative adjuvant therapy was not significant for improving prognosis, only the PFS had slight improvement (Figure 2C and D). These results suggested that total resection of tumors and active adjuvant radiotherapy and chemotherapy after operation have positive effects on improving the clinical outcomes of sGBM patients.

### Treatment Outcomes for Patients With Different IDH1 Mutation Status

**Figure 3.**
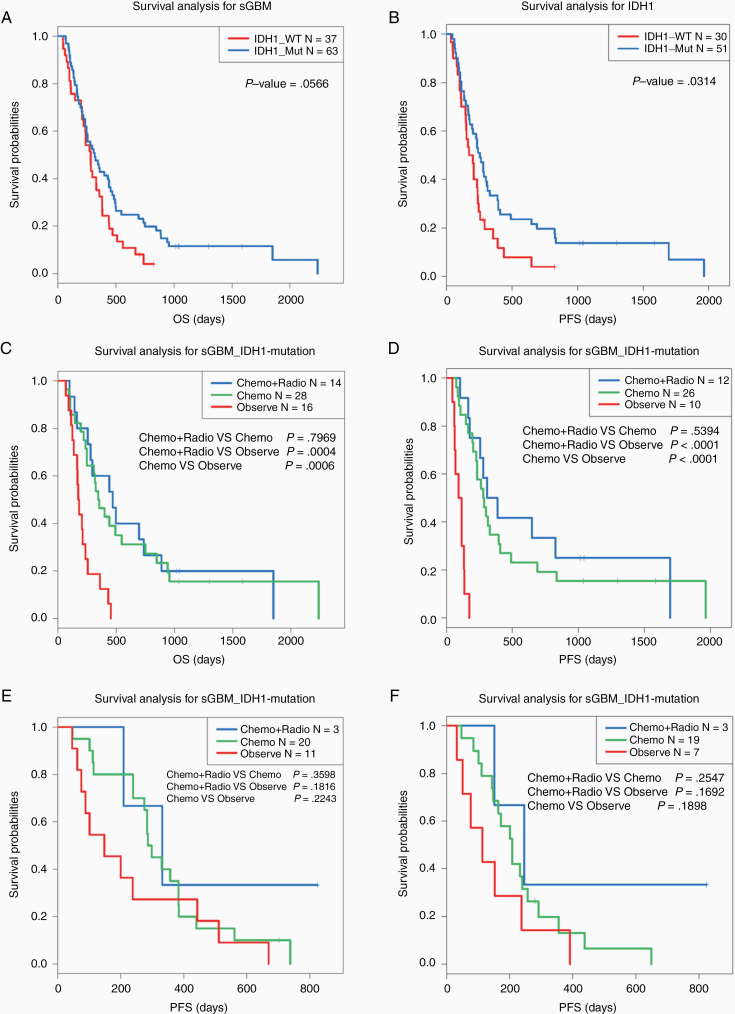
Kaplan–Meier survival curve analysis for sGBM patients with different IDH1 mutation status. (A and B) OS and PFS between IDH1 mutations and wildtype groups in sGBM patients. (C and D) OS and PFS among IDH1-mutated sGBM patients with different postoperative adjuvant therapy. (E and F) OS and PFS among IDH1-wildtype sGBM patients with different postoperative adjuvant therapy.

It is generally known that the IDH1mutation has a significant effect on the prognosis of glioma patients.^14^ However, patients with IDH1 mutations only showed a better PFS compared with IDH1 wildtype patients in sGBM (Figure 3A and B). After dividing sGBM patients into 2 subgroups according to IDH1 mutation status, we found that, in patients with IDH1 mutations, postoperative adjuvant chemotherapy combined with radiotherapy or chemotherapy alone can significantly improve clinical outcomes of patients (Figure 3C and D). However, no similar results were found in IDH1 wildtype patients (Figure 3E and F). These results implied that patients with IDH1 mutation can benefit significantly from postoperative adjuvant therapy.

### COX Regression Analyses of the Clinicopathological and Molecular Features

Univariate Cox regression analysis was performed to further determine the prognostic factors of sGBM (Tables 2 and 3). The results suggested that postoperative adjuvant therapy was a prognostic factor for patients with sGBM (*P* < .001 for OS and PFS). Furthermore, IDH1 mutation status, preoperative KPS, and EOR were also have prognostic value for patients with sGBM. Then, a multivariate Cox regression analysis was performed incorporating IDH1 mutation status, postoperative adjuvant therapy, preoperative KPS, and EOR (Tables 2 and 3). The results revealed that only postoperative adjuvant therapy was an independent predictive factor for the OS and PFS of patients with sGBM (*P* = .001 for OS and *P* = .005 for PFS) and EOR was only an independent predictive factor for OS (*P* = .009 for OS).

**Table 2.** Univariate and Multivariate Analysis of OS in sGBM Patients

Variables	Univariate analysis		Multivariate analysis	
	HR (95% CI)	*P* value	HR (95% CI)	*P* value
Age	0.980 (0.960–1.000)	.053		
Gender	1.248 (0.875–1.779)	.222		
IDH1 mutation	0.656 (0.424–1.016)	.059		
1p19q-codel	0.577 (0.282–1.184)	.134		
MGMT-methy	1.159 (0.638–2.105)	.627		
Preoperative KPS	0.990 (0.976–1.004)	.172		
Postoperative KPS	0.978 (0.963–0.992)	.003	1.016 (0.994–1.038)	.150
EOR	0.487 (0.347–0.682)	<.001	0.602 (0.411–0.882)	.009
Adjuvant therapy	0.396 (0.260–0.603)	<.001	0.332 (0.174–0.633)	.001

CI, confidence interval; HR, hazard ratio.

**Table 3.** Univariate and Multivariate Analysis of PFS in sGBM Patients

Variables	Univariate analysis		Multivariate analysis	
	HR (95% CI)	*P* value	HR (95% CI)	*P* value
Age	0.990 (0.970–1.011)	.340		
Gender	1.200 (0.814–1.770)	.358		
IDH1 mutation	0.590 (0.362–0.961)	.034	0.828 (0.484–1.415)	.490
1p19q-codel	0.663 (0.309–1.424)	.292		
MGMT-methy	0.972 (0.515–1.835)	.931		
Preoperative KPS	0.996 (0.979–1.013)	.608		
Postoperative KPS	0.970 (0.953–0.988)	.001	1.003 (0.971–1.036)	.864
EOR	0.680 (0.465–0.993)	.046	0.790 (0.458–1.362)	.397
Adjuvant therapy	0.279 (0.169–0.460)	<.001	0.253 (0.098–0.654)	.005

CI, confidence interval; HR, hazard ratio.

## Discussion

Although sGBM and pGBMs have a similar histological appearance,^5^ the sGBM patients usually have better clinical outcomes compared with patients with pGBM.^9,15^ Meanwhile, the predisposing age of sGBM is much younger than pGBM. To our knowledge, the status of IDH1 mutation, MGMT promoter methylation, and 1p19q codeletion have a significant impact on the prognosis of glioma and this conclusion also applies to pGBM.^16–18^ However, we found that these key molecular markers did not have similar prognostic value in sGBM (Table 1). Therefore, we have reason to believe that pGBM and sGBM are distinct tumor entities and may require different therapeutic approaches.

As sGBM only accounts for a small portion of GBM,^19^ few studies have focused on the treatment of sGBM. Therefore, the clinical and biological characteristics of sGBM remain unclear, and there is still no reliable evidence for the treatment of sGBM. In 1 study by SongTao Q et al. showed that IDH1/2 mutation, MGMT promoter methylation, and 1p19q codeletion were independent prognostic factors in sGBM.^10^ However, the above study did not include a complete postoperative adjuvant treatment for survival analysis. Our study found postoperative adjuvant therapy has a strong prognostic value compared with other factors. Two other researches by German teams included complete clinical data and molecular pathology information for prognostic analysis of sGBM.^11,12^ However, the number of patients included in the 2 studies was 45 and 39, respectively. The limited sample size limited the accuracy and stability of their results. We have established the largest cohort of sGBM patients to date and found that only postoperative adjuvant therapy and EOR were prognostic factor for sGBM. Although patients with IDH1 mutation showed more benefit from adjuvant therapy after surgery, IDH1 mutation status was not the independent prognostic factors of sGBM patients. The most likely reason is IDH1 mutation status changes the clinical outcome of sGBM patients by affecting the effectiveness of postoperative adjuvant therapy. Therefore, the significance of maximum resection and molecular pathology in sGBM treatment should not be ignored. However, all results need to be further validated in a larger cohort of patients and high-quality prospective studies.

In previous studies, we identified PTPRZ1-MET (ZM) fusion as a recurrent fusion gene and investigated the function of ZM fusion in sGBM. We found that ZM fusion played an active role in the development of sGBM.^20^ In this study, ZM fusion-positive patients account for 27% of all sGBM patients, total surgical resection and active postoperative adjuvant therapy cannot improve the poor prognosis of these patients (Supplementary Figure S2). Meanwhile, ZM fusion-negative patients can benefit from active postoperative adjuvant therapy (Supplementary Figure S3). According to our previous clinical trials on the small molecular inhibitor named PLB-1001,^4^ we believed that ZM fusion-positive patients may sensitive to MET inhibitors.

In conclusion, an active postoperative adjuvant therapy and gross total resection were independent positive prognostic factor for sGBM. Moreover, the requirement for full benefit from adjuvant therapy was IDH1 mutation.

## Conclusion

In this study, we investigated the prognostic factors and treatment decisions of patients with sGBM. The results suggested that gross total resection combine with postoperative radiotherapy and chemotherapy could significantly improve clinical outcome of patients with sGBM. However, we also found that this treatment strategy was not suitable for IDH1-wildtyped patients. Thus, we recommended aggressive postoperative adjuvant therapy after gross total resection to most patients with sGBM. For IDH1-wildtyped patients, more in-depth researches and novel treatment strategies are needed.

## Supplementary Material

vdaa098_suppl_Supplementary_Material

## Notes

**Authorship Statement.** Designed study: Z.Z. and T.J. Wrote the paper: RH and GL. Statistical analysis: C.Z. and Z.W. Prepared figures and contributed to writing: Y.L. and W.Z. Case follow-up and data collation: Y.W., P.Y., and D.Z.
