# Supplementary material for: Long-term efficacy of surgical resection with or without adjuvant therapy for treatment of secondary glioblastoma in adults
Source: Neurooncol Adv. 2020 Aug 21;2(1):vdaa098. doi: 10.1093/noajnl/vdaa098 (PMC7513886; doi:10.1093/noajnl/vdaa098)
Supplement: vdaa098_suppl_Supplementary_Material [file vdaa098_suppl_supplementary_material.docx]

**Supplementary Materials**

**
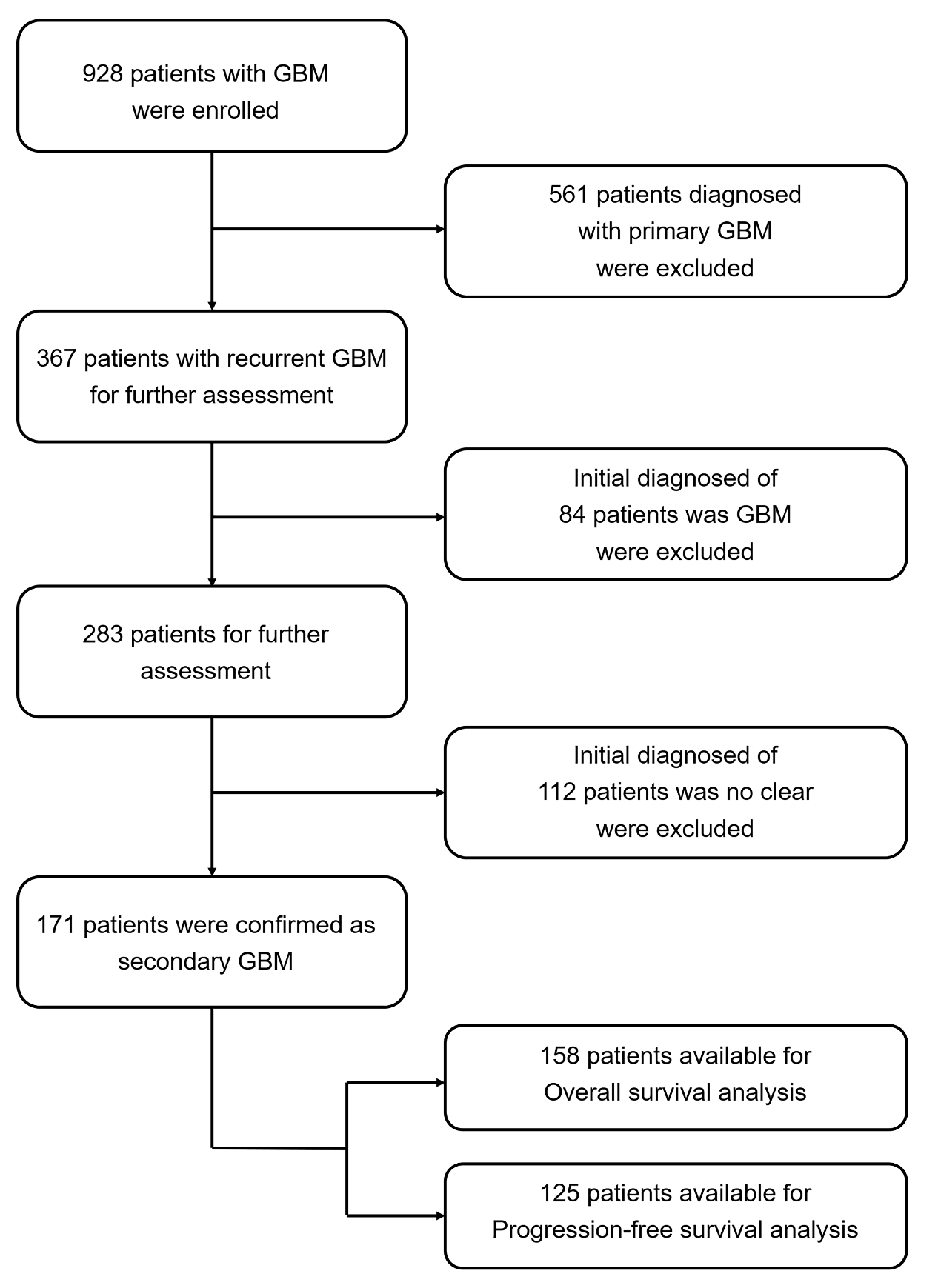
Supplementary Figure 1.** Flow diagram of sGBM patients. Patient information was obtained from CGCG database, 171 sGBM patients eligible for the study were screened from the sample of 928 GBM patients.


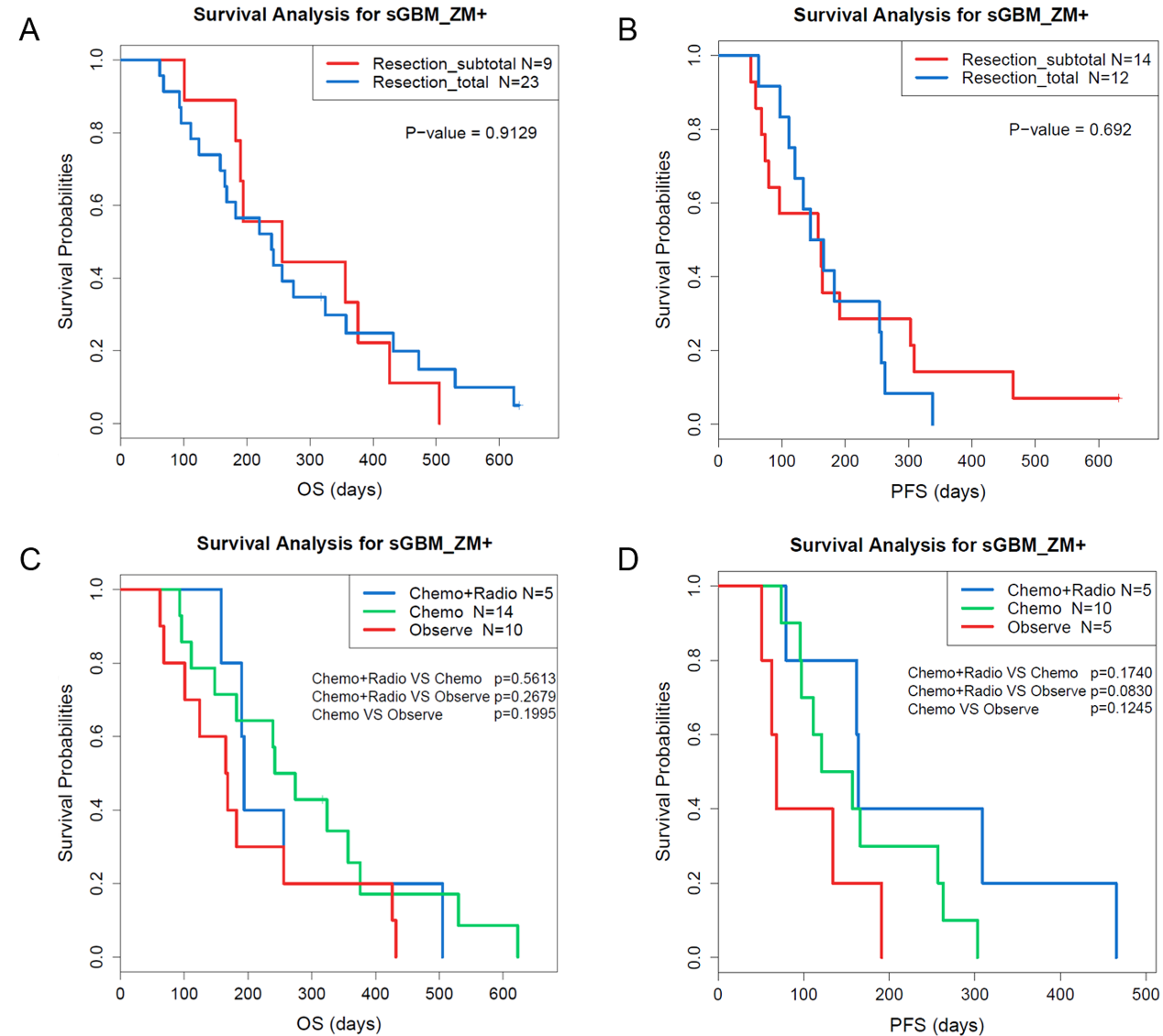


**Supplementary Figure 2.** Kaplan–Meier survival curve analysis for sGBM patients with PTPRZ1-MET (ZM) fusion. (**A, B**) OS and PFS between ZM fusion-positive sGBM patients with different extent of resection. (**C, D**) OS and PFS among ZM fusion-positive sGBM patients with different postoperative adjuvant therapy.


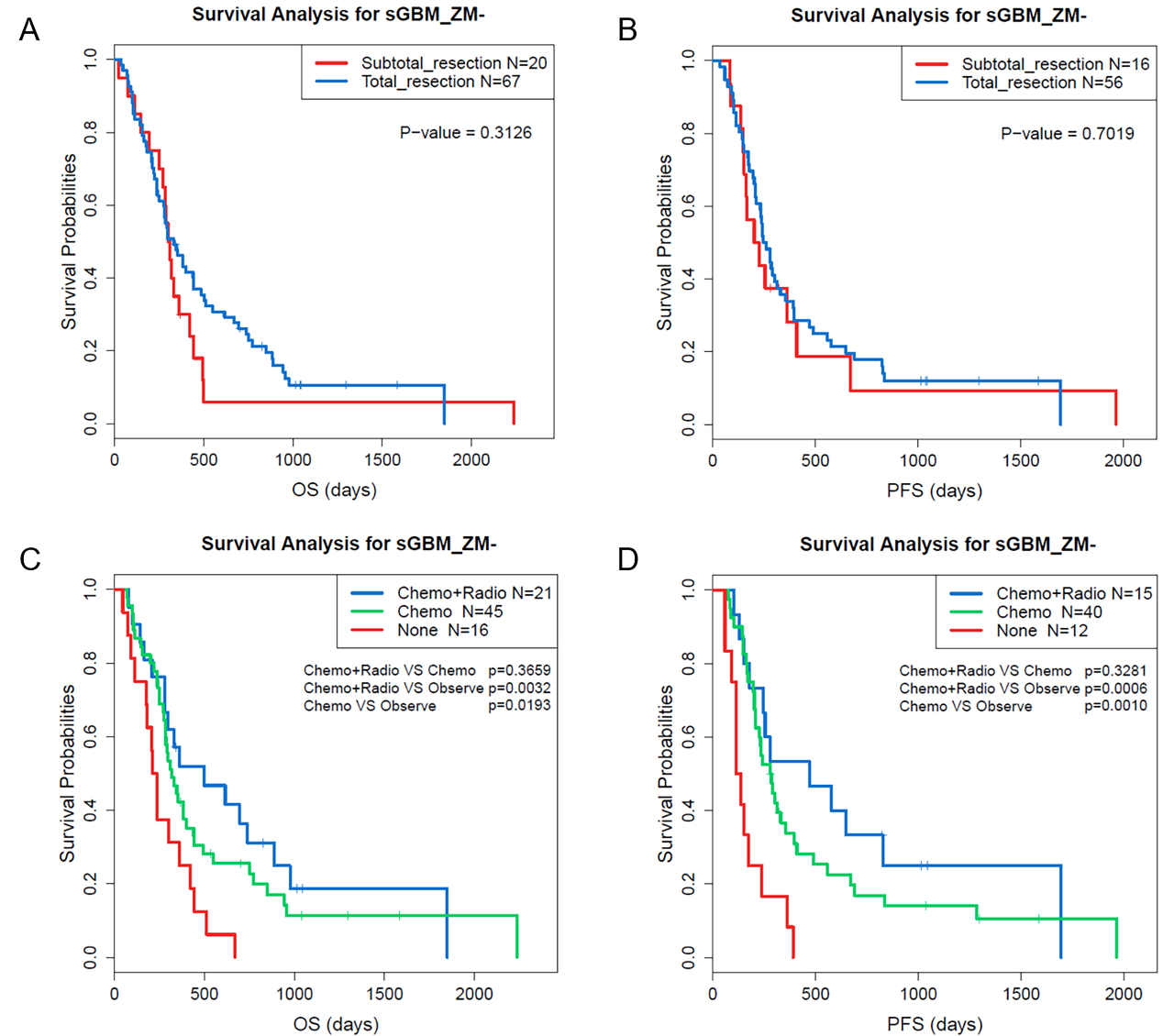


**Supplementary Figure 3.** Kaplan–Meier survival curve analysis for sGBM patients without PTPRZ1-MET (ZM) fusion. (**A, B**) OS and PFS between ZM fusion-negative sGBM patients with different extent of resection. (**C, D**) OS and PFS among ZM fusion- negative sGBM patients with different postoperative adjuvant therapy.

| **Supplementary Table 1. Association between resection level and clinical characteristics** | | | | |
| --- | --- | --- | --- | --- |
| Characteristic | | Total-resection | Subtotal-resection | p value |
| Gender | |  |  | 0.6280 |
|  | Male | 59 | 54 |  |
|  | Female | 33 | 25 |  |
| Age (years) | |  |  | >0.9999 |
|  | ≤40 | 43 | 36 |  |
|  | ＞40 | 49 | 43 |  |
| Postoperative treatment | |  |  | 0.1546 |
|  | Chemo+Radio | 22 | 12 |  |
|  | Chemo | 40 | 37 |  |
|  | Observe | 15 | 21 |  |
| IDH1 Status | |  |  | 0.5467 |
|  | Mutation | 41 | 26 |  |
|  | Wildtype | 22 | 19 |  |
| MGMT Promoter | |  |  | 0.3910 |
|  | Methylation | 19 | 15 |  |
|  | Unmethylation | 14 | 6 |  |
| 1p19q | |  |  | 0.1392 |
|  | Codel | 8 | 1 |  |
|  | Intact | 34 | 23 |  |
| KPS | |  |  |  |
|  | Preoperative KPS | 87 | 78 | 0.106* |
|  | Postoperative KPS | 92 | 79 | 0.353* |

*Wilcoxon Signed Ranks Test
